# Critical gaps in laboratory leadership to meet global health security goals

**DOI:** 10.2471/BLT.17.195883

**Published:** 2017-08-01

**Authors:** Adilya Albetkova, Jocelyn Isadore, John Ridderhof, Renee Ned-Sykes, Lucy Maryogo-Robinson, Eric Blank, Sebastien Cognat, Virginie Dolmazon, Philippe Gasquet, Mark Rayfield, Leonard Peruski

**Affiliations:** a Division of Global Health Protection, Centers for Disease Control and Prevention, Atlanta, GA 30329, United States of America (USA).; b Association of Public Health Laboratories, Silver Spring, USA.; c Division of Laboratory Systems, Centers for Disease Control and Prevention, Atlanta, USA.; d World Health Organization Lyon Office, Lyon, France.

Public health laboratories play a critical role in the detection, prevention and control of diseases. However, reliable laboratory testing continues to be limited in many low- and middle-income countries.^1^ The 2013–2016 Ebola virus disease outbreak in West Africa provided many examples of how functioning laboratories were needed for disease control and prevention efforts.^2^ This outbreak highlighted the need for laboratory directors to be able to influence national laboratory policy and to implement national laboratory strategic plans.^3^^,^^4^ Global health initiatives such as The United States President’s Emergency Plan for AIDS Relief (2003),^5^ the *International Health Regulations* (IHR; 2005),^6^ the Global Health Security Agenda (GHSA; 2014)^5^ and the health-related United Nations sustainable development goal (SDG 3) of the *2030 Agenda for sustainable development* (2015),^7^ all emphasize the need for laboratory systems capable of providing affordable, sustainable and quality laboratory testing. However, despite progress made, only 22% (42/193) of countries reported meeting the IHR core capacities’ requirements for surveillance and response by the June 2012 target date and 34% (65/193) by the November 2015 target date.^5^^,^^6^ The GHSA was launched in 2014 to accelerate progress towards global health preparedness and to support capacity-building efforts. The GHSA objectives and IHR’s core capacities overlap in several areas, including laboratory systems and workforce development.^5^

The GHSA Workforce Development Action Package^8^ outlines the need for rigorous and sustainable training programmes for public health professionals and emphasizes the need for practical, hands-on experience to support public health systems. Ideally, such programmes would help the public health laboratory workforce in gaining the skills and expertise to navigate an often-chaotic environment.^9^

The World Health Organization (WHO) has long articulated the need for specialized training for laboratory directors in the areas of leadership and management. Other initiatives have emerged, but they only partially address the identified needs. The Centers for Disease Control and Prevention’s (CDC’s) Laboratory Leadership Service^10^ is a new fellowship programme that provides a comprehensive competency-based leadership training in the United States. The European Centre for Disease Prevention and Control also offers the European Union Public Health Microbiology Training Programme,^11^ a fellowship which includes a leadership and management training focused on Europe. The American Society for Microbiology (ASM)/CDC Program for Infectious Disease and Public Health is a postdoctoral fellowship programme^12^ that strives to provide fellows with in-depth and quality training on management in support of improving the quality of health services; however, the management and leadership content in the programme is limited. The International Training and Education Center for Health offers a certificate programme in laboratory leadership and management, but to date, it has been implemented only in 10 countries in the WHO Eastern Mediterranean region.^13^ These fellowship and training programmes offer varied approaches that aim at reinforcing national laboratory systems through workforce development and have provided the first steps towards strengthening competency attainment^14^ in public health laboratory leaders. All initiatives point to the need for a comprehensive global framework, including competency-based and laboratory-specific leadership training as well as a mentorship approach.

In this context, WHO, CDC and the Association of Public Health Laboratories have agreed to define leadership competencies, based on CDC’s and the Association of Public Health Laboratories’ competency guidelines,^14^ and to develop a Global Laboratory Leadership Program to address gaps in the education and training of laboratory directors. The Global Laboratory Leadership Program is a fellowship programme that will use a standard curriculum and implementation framework designed to transform mid-level laboratory managers and scientists into effective leaders for a critical component of global health security.
